# 653. Direct Identification of Microorganisms in Positive Blood Cultures by the BioFire® FilmArray® Blood Culture Identification Panel Leads to Faster Optimal Antibiotic Therapy: A Before–After Study

**DOI:** 10.1093/ofid/ofab466.850

**Published:** 2021-12-04

**Authors:** Jessica Agnetti, Andrea C Büchler, Michael Osthoff, Fabrice Helfenstein, Vladimira Hinic, Sarah Tschudin-Sutter, Veronika Bättig, Nina Khanna, Adrian Egli

**Affiliations:** 1 University of Basel, Basel-Stadt, Switzerland; 2 Erasmus University Medical Center, Rotterdam, Zuid-Holland, Netherlands; 3 University Hospital Basel, Basel-Stadt, Switzerland; 4 University hospital Basel, Basel-Stadt, Switzerland

## Abstract

**Background:**

Rapid pathogen identification from positive blood cultures may help optimize empiric antibiotic therapy quickly by reducing unnecessary broad spectrum antibiotic use and may improve patient outcomes. The BioFire® FilmArray® Blood Culture Identification Panel 1 (BF-FA-BCIP) identifies 24 pathogens directly from positive blood cultures without subculture. 3 resistance genes are included. We aimed to compare the time to optimal antibiotic therapy between BF-FA-BCIP and conventional identification.

**Methods:**

We performed a single-center retrospective case-control before-after study of 386 cases (November 2018 to October 2019) with BF-FA-BCIP compared to 414 controls (August 2017 to July 2018) with conventional identification. The primary study endpoint was the time from blood sampling to implementation of optimal antimicrobial therapy. Secondary endpoints were time to effective therapy, length of hospital stay, and in-hospital and 30-day mortality. Outcomes were assessed using cause-specific Cox Proportional Hazard models and logistic regressions.

**Results:**

We included 800 patients with comparable baseline characteristics. Main sources of blood stream infection (BSI) were urinary tract infection and intra-abdominal infection (19.2% vs. 22.0% and 16.8% vs. 15.7% for case and control groups, respectively). Overall, 212 positive blood cultures were considered as contaminations. Identification results were available after a median of 21.9 hours by the BF-FA-BCIP and 44.3 hours by the conventional method. Patients with BF-FA-BCIP received the optimal therapy after a median of 25.5 hours (95%CI 21.0 - 31.2) as compared to 45.7 hours (95%CI 37.7 - 51.2) in the control group (Figure 1). We found no effect of the identification method on secondary outcomes.

Kaplan-Meier curve representing the probability of implementing the optimal therapy at any given time according to the identification method (Standard vs. BF-FA-BCIP).

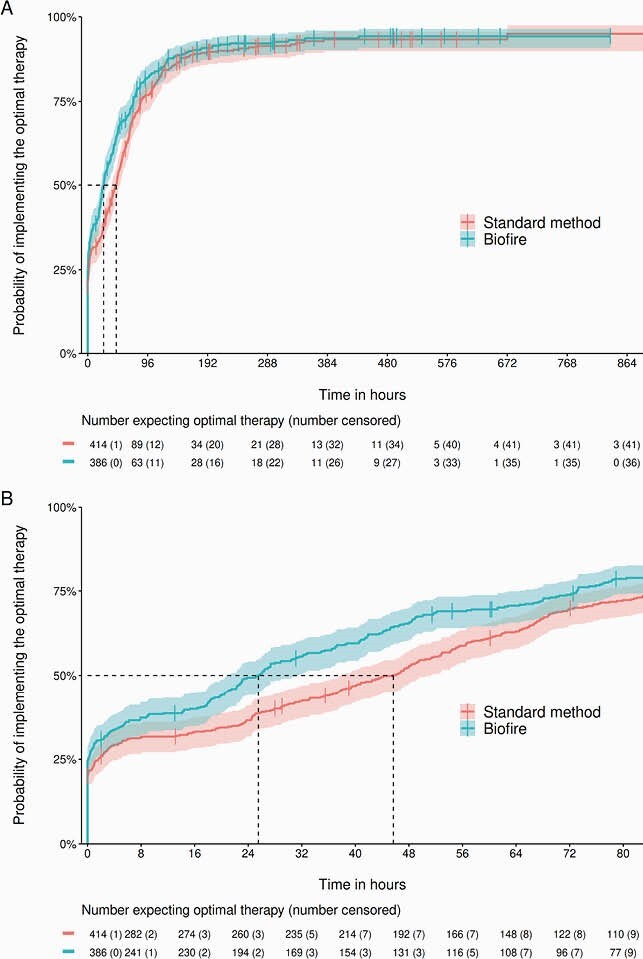

Shaded ribbons represent the 95 % confidence interval (CI). The vertical dashes represent censored data. The vertical dotted lines represent the median time, i.e. the time at which 50 % of the patients obtained the optimal therapy, for the two methods. Median (95 % CI) time to optimal therapy is 45.7 (37.7 - 51.4) hours with the Standard method and 25.5 (21.0- 31.2) hours with Biofire. The tables below the curves present the numbers expecting optimal therapy according to the bacteria identification method, as well as the number of censored data in parenthesis. Panel A shows data from 0 to 900 hours. Panel B shows the data from 0 to 90 hours to better visualize how the probability to implement optimal therapy varies in the first 72 hours.

**Conclusion:**

In conclusion, rapid pathogen identification by BF-FA-BCIP was associated with an almost 24h earlier initiation of the optimal antibiotic therapy in BSI. However, the overall benefit for individual patients seems to be limited. Future studies should assess the cost-effectiveness and impact on the prevention of antibiotic resistance using this diagnostic approach.

**Disclosures:**

**All Authors**: No reported disclosures

